# Age of Diagnosis, Fidelity and Acceptability of an Early Diagnosis Clinic for Cerebral Palsy: A Single Site Implementation Study

**DOI:** 10.3390/brainsci11081074

**Published:** 2021-08-16

**Authors:** Anna te Velde, Esther Tantsis, Iona Novak, Nadia Badawi, Jane Berry, Prue Golland, Johanna Korkalainen, Robyn McMurdo, Ronda Shehata, Catherine Morgan

**Affiliations:** 1 Cerebral Palsy Alliance Research Institute, Specialty of Child and Adolescent Health, Sydney Medical School, Faculty of Medicine and Health, The University of Sydney, Sydney, NSW 2006, Australia; inovak@cerebralpalsy.org.au (I.N.); nadia.badawi@health.nsw.gov.au (N.B.); cmorgan@cerebralpalsy.org.au (C.M.); 2 Specialty of Child and Adolescent Health, Sydney Medical School, Faculty of Medicine and Health, The University of Sydney, Sydney, NSW 2006, Australia; 3 TJ Nelson Department of Neurology and Neurosurgery, Children’s Hospital Westmead, Westmead, NSW 2145, Australia; esther.tantsis@health.nsw.gov.au; 4 Faculty of Medicine and Health, The University of Sydney, Sydney, NSW 2006, Australia; 5 Grace Centre for Newborn Intensive Care, Sydney Children’s Hospital Network, The University of Sydney, Westmead, NSW 2145, Australia; 6 Cerebral Palsy Alliance, Allambie Heights, NSW 2100, Australia; jberry@cerebralpalsy.org.au (J.B.); pgolland@cerebralpalsy.org.au (P.G.); jkorkalainen@cerebralpalsy.org.au (J.K.); rmcmurdo@cerebralpalsy.org.au (R.M.); rshehata@cerebralpalsy.org.au (R.S.)

**Keywords:** cerebral palsy, early diagnosis, implementation science, knowledge translation, infant, early intervention

## Abstract

Cerebral palsy (CP) diagnosis is historically late, at between 12 and 24 months. We aimed to determine diagnosis age, fidelity to recommended tests and acceptability to parents and referrers of an early diagnosis clinic to implement a recent evidence-based clinical guideline for the early diagnosis of CP. A prospective observational case series of infants <12 months with detectable risks for CP attending our clinic was completed with data analysed cross-sectionally. Infants had a high risk of CP diagnosis at a mean age of 4.4 (standard deviation [SD] 2.3) months and CP diagnosis at 8.5 [4.1] months. Of the 109 infants seen, 57% had a diagnosis of CP or high risk of CP, showing high specificity to our inclusion criteria. Parent and referrer acceptability of the clinic was high. Paediatricians had the highest rate of referral (39%) followed by allied health (31%), primary carer (14%) and other health workers (16%). Fidelity to the guideline was also high. All infants referred <5 mths had the General Movements Assessment (GMA) and all except one had the Hammersmith Infant Neurological Examination (HINE) administered. *N* = 92 (84%) of infants seen had neuroimaging, including *n* = 53 (49%) who had magnetic resonance imaging (MRI), showing recommended tests are feasible. Referral to CP-specific interventions was at 4.7 [3.0] months, sometimes before referral to clinic. Clinicians can be confident CP can be diagnosed well under 12 months using recommended tools. This clinic model is acceptable to parents and referrers and supports access to CP-specific early interventions when they are likely to be most effective.

## 1. Introduction

Cerebral palsy (CP) is diagnosed between 12 and 24 months in high-income countries [1,2] and later in low- and middle-income countries, for example, 5 years in Bangladesh [3]. A recent international clinical practice guideline (hereafter termed “guideline”) outlines 12 recommendations, including the best evidence-based assessments, to make an accurate and early diagnosis of CP at 3–12 months of age [1]. The guideline recommends an interim “at high risk of CP” diagnosis to accelerate identification in mild or ambiguous cases. Early diagnosis enables access to CP-specific evidence-based [4] interventions at an age when the infant brain has the most neuroplastic potential [5]. The guideline has been implemented in some American follow up services, lowering the age of diagnosis [6,7].

The guideline outlined two early detection pathways for the most accurate diagnosis depending on age. Infants less than 5 months old are assessed using the “newborn detectable risks” pathway, which recommends the General Movements Assessment (GMA), brain magnetic resonance imaging (MRI) and the Hammersmith Infant Neurological Examination (HINE), a standardised neurological exam [1]. Infants older than 5 months are assessed on the “infant detectable risks” pathway, in which HINE, MRI and standardised motor assessments are recommended [1], since the GMA is no longer valid. The guideline provides predictive validity for multiple standardised assessments and recommends which motor assessment to use in each age epoch; for example, the parent-reported Developmental Assessment of Young Children (DAYC) [8] is conditionally recommended for use in the infant risks group where parents are often identifying concerns. Identifying all infants with CP early requires comprehensive ascertainment because multiple causal pathways and timing of injury exist [9,10]. Newborn Intensive Care Unit (NICU) graduates with identifiable risks for CP are often followed in outpatient clinics [11] on the newborn risks pathway. However, NICU follow-up criteria vary, meaning cases are missed. Infants not meeting these criteria or those with an uncomplicated birth are typically referred later to a neurologist or paediatrician after parents or a professional identify concerns, e.g., milestone delays or asymmetries, and are usually assessed on the infant risks pathway, meaning diagnosis is made later.

Context-specific, multifaceted knowledge translation (KT) approaches should be paired with guidelines for successful implementation to close the known 15–20 year knowledge to practice gap [12]. Our group has employed country-wide knowledge translation interventions to lower the age of diagnosis. In stage one, we promoted the uptake of recommended tools (GMA and HINE) as the new standard of care by: (1) establishing a community of practice for reliability and use of the GMA; (2) educating opinion leaders; (3) seeking two Australian GMA trainers to enable local training; (4) providing financial scholarships to key opinion leaders/brokers. These stage one KT strategies improved use of the GMA (and HINE) in NICUs, as evidenced by published data on the GMA’s predictive accuracy [13]. However, gaps in early diagnosis persisted, including: (1) clinician confidence to make an early diagnosis; and (2) absence of diagnostic services for infants not meeting NICU follow-up criteria or with no known risks for CP at birth or with no neurology services available. In a stage two response, we established a specialist early diagnosis clinic, hereafter termed “clinic”, to expedite diagnosis in the community. This paper reports on the impact of the clinic in lowering the age of diagnosis in both NICU and non-NICU graduates.

The primary aim of this paper was (1) to determine age of diagnosis of infants attending our clinic. Secondary aims were to determine: (2) if the infant risks group are diagnosed later than the newborn risks group; (3) if the right infants were targeted for the diagnostic process; (4) if guideline fidelity could be maintained [1]; and (5) acceptability of the clinic by parents and referrers. We hypothesised: (1) infants could be diagnosed under 12 months by implementing guideline recommendations underpinned by a multifaceted KT strategy; (2) the infant risks group would be diagnosed later than the newborn risks group; (3) >50% of infants referred would have CP or high risk of CP based on clinic eligibility criteria; (4) fidelity to evidence pathways in the guideline [1] could be maintained; and (5) parents and referrers would find the clinic acceptable.

## 2. Materials and Methods

### 2.1. Design

A prospective observational case series was conducted. Data for the first 30 months of clinic operation, from March 2018, were analysed cross-sectionally. No sample size was calculated, with all eligible infants included. Results were reported as per the Standards for Reporting Implementation Studies statement [14] (Supplementary Table S1).

### 2.2. Implementation Strategy

**Figure 1:**
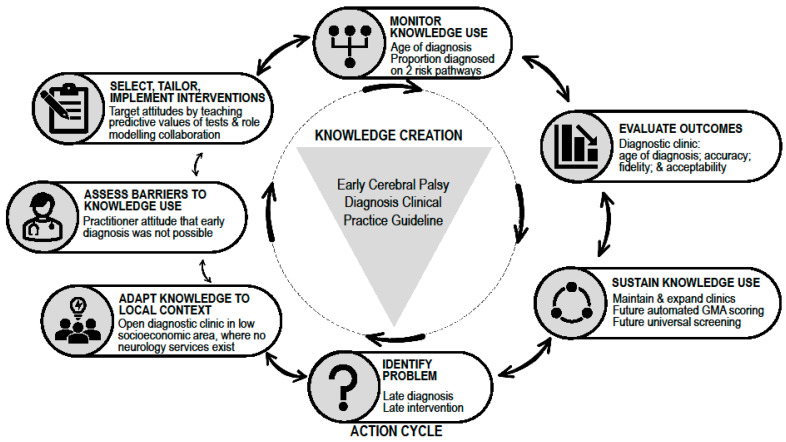
Knowledge translation strategy example using Knowledge-to-Action cycle [25]. Abbreviation: GMA, General Movements Assessment.

We applied a multifaceted knowledge translation package to our newly established diagnostic clinic to lower the age of diagnosis in the community. The clinic structure is outlined in Table 1. Eight evidence-based KT strategies were selected as solutions to overcome barriers to guideline implementation [15,16], including: (1) targeting practitioner attitudes regarding early diagnosis [17]; (2) guideline awareness [18]; (3) knowledge brokers’ use of early diagnosis evidence to inform policymakers within both collaborating institutions; (4) use of opinion leaders [19]; (5) industry experts mentoring clinic staff [20]; (6) medical and parent self-referral to clinic (parent-mediated KT strategy) [21]; (7) leveraging on a research active culture, including employing research clinicians [22]; and (8) Neurologist employed as medical lead [23]. KT strategy planning utilised the Knowledge-to-Action process [24] (Figure 1). Supplementary Table S2 details all identified barriers, facilitators and supporting evidence for KT strategies selected.

### 2.3. Implementation Site

Referrals to clinic were primarily from one state, New South Wales, with a population of 8.16 million and 315,000 births annually [26], and targeted a region with a growing population and high level of socioeconomic disadvantage, due to the socioeconomic gradient associated with disability [27].

### 2.4. Clinic Eligibility Criteria

Eligibility criteria were designed to capture any infant with risks for CP but not yet diagnosed. These included:

1. <12 months corrected age; and
2. No current neurologist; and
3. Medical referral; and
4. Signs of motor dysfunction, for example:
  Trajectory of cramped synchronised general movements at writhing age, absent fidgety general movements at fidgety age [28]; or
  - Below average scores on standardised motor assessment; or
  - Specific motor milestones delay, e.g., hand asymmetry >4 months, not sitting >9 months [29] and either:
5. Clinical history with risks for CP [1,30,31]; or
6. Neuroimaging indicating motor impairment [1].

### 2.5. Outcomes

#### 2.5.1. Primary Outcomes

Age of diagnosis (guideline recommendation 1.0) was analysed at three “stages” in the diagnosis process: (1) age any service suspected CP, as per the guideline criteria; (2) age when CP or high risk of CP diagnosis given at clinic; and (3) age CP confirmed. Diagnostic outcomes included (1) CP [32]; (2) high risk of CP [1]; (3) no CP, other diagnosis; and (4) no CP, no apparent concerns.

#### 2.5.2. Secondary Outcomes

Fidelity to guideline recommendations was measured using the proportion of infants with (1) diagnostic tests completed (recommendations 2.0–6.2); (2) referral to CP-specific early intervention, disability-specific funding and CP-specific medical services (recommendation 10.0); and (3) screened for associated impairments (recommendation 11.0).

Acceptability of parents and referrers was measured using (1) referrer profiles; (2) proportion of appointments attended; and (3) Measure of Processes of Care (MPOC-20) questionnaires [33], which measure family centeredness of a service on five scales from 0 (not at all) to 7 (to a very great extent). Questionnaires were completed by anonymous mail survey.

### 2.6. Statistical Analysis

Outcomes were analysed using descriptive statistics. All analyses were completed in Excel^®^ 2019. Independent t-test was used to analyse between group differences for age of diagnosis.

## 3. Results

### 3.1. Eligibility, Age of Diagnosis and Diagnosis Outcomes

Eligibility, age of referral: Infant demographics and risk factors are reported in Table 2. Of *n* = 148 infants referred, *n* = 109 had attended, *n* = 12 were waiting on an appointment, *n* = 2 declined or not contactable and *n* = 25 were ineligible. The *n* = 109 infants seen were referred at mean age 5.7 (standard deviation [SD] 3.1) months and first seen at 7.2 [3.3] months.

Age of diagnosis (Table 3): Of the n = 109 infants assessed, the mean age any referring service suspected CP was 4.4 [2.3] months. CP diagnosis was confirmed in clinic at 8.5 [4.1] months. The newborn risk group was identified earlier for suspected CP (3.6 [1.1] months) than the infant detectable risks group 9.0 [2.7] months (*t* = −5.25, *p* = 0.00), but no difference was found at age of CP diagnosis (newborn detectable risks group 8.1 [4.2] months; infant detectable risks group 10.6 [1.9] months, *t* = −2.12, *p* = 0.05).

Diagnostic outcome (Table 4): A CP or high risk of CP diagnosis was given to 57% of infants, accounting for 19% of infants estimated to be diagnosed with CP in New South Wales during the study period. One infant (1%) had CP diagnosis, which was later revoked and for analysis is included in the no CP, other diagnosis group.

### 3.2. Fidelity to International Clinical Guideline

#### 3.2.1. Diagnostic Tests

Proportions of tests completed are reported in Table 5. All infants identified <5 months were assessed with GMA. All infants, except one, had HINE completed. Neuroimaging: Of n = 109 infants seen, 84% had neuroimaging prior to clinic. n = 43 (39%) were referred for MRI from clinic. Combination of GMA, neuroimaging and HINE: In total, n = 19 infants had triangulating findings predictive of CP (i.e., absent fidgety on GMA; neuroimaging predictive of motor impairment and HINE below reported CP cut scores [35]), of whom n = 17 had a diagnosis of CP; n = 2 had global developmental delay or attention problems. Genetic testing: While not an explicit guideline recommendation, some infants were sent for genetic screening if CP aetiology was unclear, e.g., infants with normal or non-specific MRI findings or dysmorphic features.

#### 3.2.2. CP Classification

(Recommendations 8.0–9.0): Predominant motor type: Of the 48 infants with CP, 73% were classified as spastic, 15% dyskinetic, 0% ataxic, 2% hypotonic predominant motor type as per the Australian Cerebral Palsy Register [36] and 10% were too young to classify accurately. Topography: Of the n = 38 infants with spastic motor type, 47% had hemiplegia, 21% diplegia and 32% quadriplegia. Motor Severity: We used the Gross Motor Function Classification System (GMFCS) [37] to classify infants with CP (n = 48): Level I (50%), level II (25%), level III (0%), level IV (8%), and level V (8%) with 8% too young to classify as <12 months. Topography and motor severity data reflect Australian population register trends [36], suggesting a representative sample with external validity.

#### 3.2.3. Referral to CP-Specific Early Intervention Funding and Medical Services (Recommendation 10.0)

For infants with CP, 98% were referred to CP-specific early interventions, with services first accessed at 4.7 [3.0] months. All were referred for funding through the National Disability Insurance Scheme at 4.0 [4.0] months. Seventy-seven percent were referred to CP-specific medical rehabilitation services for management of tone, pain and hip surveillance at 12.1 [5.1] months.

#### 3.2.4. Associated Impairment Screening (Recommendation 11.0)

The following concerns were identified and referrals were made for infants with CP: vision (46%); hearing (17%); communication delay (69%), feeding concern (40%). Risk of seizures discussion was documented in 79% of infants with CP; 23% of infants had seizure management in place; 13% had seizures identified through clinic; 52% required no seizure management and 13% did not have seizure status documented.

#### 3.2.5. Parent Wellbeing (Recommendation 1.0)

This was discussed for every family; support options were documented in 85% of cases.

### 3.3. Acceptability

Acceptability by parents and referrers was high. n = 2 (2%) of the 123 eligible infants declined an appointment or were non-contactable. Paediatricians had highest rate of referral (39%) followed by Physiotherapists (24%), primary care giver (14%), Neonatologists (7%), Occupational Therapists (7%), Neurologists (4%), Social Workers (2%), Nurses (1%), General Practitioners (1%) and other health workers (1%). Sixty percent of referred infants were also captured in NICU follow up. Parents rated the clinic on the MPOC-20, from 23 responses (21% response rate), on respectful and supportive care (mean score 6.4 [0.6]); coordinated and comprehensive care (6.4 [0.7]); providing specific information about child (5.6 [1.6]); enabling and partnership (5.9 [1.4]); and providing general information (4.8 [1.7]). No harms or unintended effects were observed.

## 4. Discussion

Our aim was to determine the age of CP diagnosis by implementing the international clinical guideline [1] in a multifaceted evidence-based knowledge translation strategy packaged as an early diagnosis clinic. We found high risk of CP was identified at 4.4 months, early intervention started by 4.7 months and CP diagnosis was made at 8.5 months on average. Infants were referred at 5.7 months, i.e., in some cases after CP was suspected and intervention commenced, showing a delay from identification to referral. These data suggest the “high risk of CP” diagnosis proposed in the guideline expedites starting interventions in the right infants at an early age. Infants with CP on the newborn risks pathway were mainly accurately identified as high risk of CP, but not given a CP diagnosis, by community providers prior to referral to this clinic. This suggests barriers still exist, for example, confidence to make and communicate an early diagnosis. Overall, we found CP could be diagnosed well under 12 months. This is lower than previously reported, for example, 18.9 months (±12.8 months) in Canada [2]. Diagnosis age from this clinic is comparable to some clinical service models used to implement the guideline [7] and even earlier than other models [6], suggesting the eligibility criteria and knowledge translation strategies that we used were effective for guideline implementation. This is particularly important given the age of diagnosis of CP has not changed over the past three decades [36].

Commencing CP-specific early interventions in the first few months of life is likely to be key to decreasing the severity of impairments, as neuroplasticity is enhanced in the young brain. Child-active motor learning interventions to harness neuroplasticity in infants with CP are underway globally [4]. However, based on our results, a proportion of infants with CP still experience up to 5 months delay accessing intervention because they did not fit the “newborn detectable risk” profile. The infant detectable risk group was a smaller proportion of our cohort and was less likely to receive a diagnosis of CP, suggesting an under representation of children with infant detectable risks in our clinic, which indicates ongoing KT issues at a community level.

The proportion of infants in our clinic diagnosed with CP or high risk of CP (57%) was higher than a recently reported neurodevelopment follow up clinic (22% CP or high risk of CP [38]) and met our hypothesis. This difference probably reflects the specificity of our eligibility criteria. There is considerable expertise in administering GMA and HINE in Australia, particularly in NICU follow up clinics [13,39] due to previous knowledge translation work. In our cohort, over 90% of infants had GMA videos and over 50% had HINE completed prior to the early diagnosis clinic appointment in NICU follow up or community services.

We were able to maintain fidelity to each of the guideline recommendations. All infants within the age for GMA had a scorable video and all except one infant had the HINE administered on at least one occasion. Access to term equivalent age (TEA) MRI was a barrier to an early or accurate diagnosis for some infants. While almost 50% of infants had an MRI prior to their initial appointment, not all were at term equivalent age as recommended [1,40,41]. Although CP is a clinical diagnosis, and 10–15% of children with CP have seemingly normal findings on MRI [42,43,44], MRI has the second highest predictive validity for detecting CP after GMA. Barriers to TEA MRI include accessibility and neonate medical stability. In addition, major lesions predictive of CP, e.g., cystic periventricular leukomalacia [40], can be detected on CUS in preterm infants; however, CUS has lower sensitivity for detecting milder motor injuries [41]. TEA MRI would support early diagnosis and earlier classification of motor severity, type and topography of CP and associated impairments, e.g., vision impairment. Infants with CP in this cohort with triangulating findings on GMA, HINE and MRI all predictive of CP corroborated the report of the high pooled accuracy of these three tests [45]. In practice, we found use of GMA, HINE and MRI was feasible as was screening for associated impairments. The Peabody Developmental Motor Scales (PDMS-2) [46] were used as a standardised motor assessment rather than the DAYC [8]. PDMS-2 is more detailed than the DAYC, and PDMS-2 was not specifically recommended in the guideline to lower assessor burden but has moderate predictive validity [1]. The Hand Assessment of Infants (HAI) [47] is also used if unilateral CP is suspected. The HAI is a newer tool, where the psychometric data became available after guideline publication.

Parents want to know at the earliest age if their child has CP [48,49]. When a diagnosis is communicated appropriately, parents are more likely to take an active role in their child’s care [50]. For parents, “high risk of CP” and CP are considered two separate moments in the diagnosis process. Accurate use of the “high risk of CP” diagnosis using guideline criteria does and should allow access to intensive interventions while further diagnostic assessment occurs. However, a CP diagnosis should still be made at the earliest possible age, giving clarity to parents. Guideline recommendations are designed for both early and accurate diagnosis. Diagnosis accuracy ensures effective distribution of health resources. In our study, <1% of infants had a CP diagnosis revoked compared to 2.9% recently reported in Canada [51]. Analysis of larger numbers using this clinic model will determine if this low rate is maintained.

Parents overwhelmingly found the clinic acceptable. Parents who found the service online made up 14% of referrals. Direct parent referral is logical, particularly for the infant detectable risks group, because parents suspect CP before a diagnosis [52] and are the most vigilant observers of their child’s development. Parents rated the clinic as moderate or high on measures of family centeredness on the MPOC-20, with scores slightly higher than children with physical disability in Iceland [53]. “Providing general information” at clinic appointments was identified as an area for improvement. In response, the Cerebral Palsy Alliance published the Early Childhood Intervention Guide [54], which is now given to parents after a diagnosis and is available online.

Future Directions: Universal GMA testing is likely to be most impactful for early detection of CP for all infants before 6 months because GMA has the best predictive validity for CP, and automated technologies to assess GMA accurately, ethically and economically are progressing [55]. An Australian precedent exists in universal newborn hearing screening, leading to earlier hearing interventions with improved hearing and language outcomes [56,57]. Human services will be required to wrap around universal GMA screening to make timely diagnoses and ensure access to early interventions. KT strategies to increase the confidence of community providers to make and communicate a diagnosis could further decrease the age of diagnosis. The proportion of infants captured in this clinic shows the scalability of the model and this clinic will be replicated in Australia. Testing of the model in various settings with site-specific barrier analysis will provide more confidence in the model and make it broadly applicable.

Limitations: This study was a single site, prospective observational case series with no comparison group available to determine the impact of the clinic compared to other models on age of diagnosis. The study design, the specificity of the location and the tailored KT strategies are limitations for the application of the model in other socioeconomic contexts. It is too early to say if this clinic model can decrease the age of diagnosis on a population level, with our inclusion criteria making this clinic more likely to diagnose CP. The Knowledge Translation of Early Cerebral Palsy study is underway in Australia testing the effectiveness of implementing recommended diagnostic tools; we await the results.

This clinic was located in a high-income country metropolitan location. Families travelled from regional locations to this clinic, showing parents want this service. Implementation strategies for remote regions and low- and middle-income country contexts are needed to ensure equal access to diagnostic services.

## 5. Conclusions

Clinicians can be confident that cerebral palsy can be diagnosed well under 12 months using the tools outlined in the guideline within the real world. Parents and referrers found our clinic model acceptable, providing another guideline implementation strategy. Infants with CP with a seemingly normal birth history are still diagnosed later and may be missing the window for early intervention under 6 months when neuroplasticity is highest. 

## Figures and Tables

**Table 1:** Description of clinic based on Template for Intervention Description and Replication (TIDieR [25]).

Name	Cerebral Palsy Alliance and NSW Health Early Diagnosis Clinic
Why	**Clinic purposes:** Decrease the age of diagnosis of CP Referral to CP-specific infant early interventions, parent wellbeing supports and funding Decrease motor severity of CP through early monitoring of associated impairments
What	**Materials:** Comfortable room with one-way viewing window and sound for observation Assessment tools: bench, weight and length measures, reflex hammer, paper tape measure, ophthalmoscope, blood pressure kit, infant toys, stairs, high chair and tray, adjustable bench Assessment kits: BSID-III, HAI, HINE and PDMS-2
	**Procedures:** Parent or health worker referral Intake officer collects infant details and relevant assessment findings Neurologist and intake officer assess eligibility A valid medical referral required for Medicare billing, the Australian universal health insurance scheme Infant, family and multidisciplinary team attend initial appointment Ongoing appointments with the purpose of diagnostic process and/or developmental surveillance to 2 years of age
Who provided	Experienced multidisciplinary team, including: neurologist, physiotherapist, occupational therapist, speech pathologist, social worker, intake officer, administration assistant and manager
How	One day/week with 4 × 90 min appointments/day Assessments, scoring, feedback to parents, liaison with other services and reporting completed on clinic day
Where	South Western Sydney, NSW, Australia. The region with highest socioeconomic disadvantage in Sydney.
When and how much	Initial appointment occurs as soon as possible after referral Ongoing appointments at 3, 6, 9, 12, 18 and 24 months corrected age as appropriate
Tailoring	Physical space is tailored as per best practice guidelines for communicating diagnoses and is a quiet, private and inviting room with enough space for staff, assessment and for both parents/support person to be present for the diagnosis
Modifications	During COVID-19 pandemic restrictions (March to June 2020), the clinic operated as a telehealth service and included the following modifications: No initial appointments No CP diagnoses made via telehealth HINE not completed via telehealth

Abbreviation: BSID-III, Bayley Scales of Infant and Toddler Development 3rd Version; CP, cerebral palsy; HAI, Hand Assessment for Infants; HINE, Hammersmith Infant Neurological Examination; NSW, New South Wales; PDMS-2, Peabody Developmental Motor Scales-2.

**Table 2:** Infant demographics and risk factors for cerebral palsy.

	All Infants, n (%) n = 109	CP, N (%) n = 48
Demographics		
Sex		
Female	46 (42)	20 (42)
Male	63 (58)	28 (58)
Prematurity		
Extreme preterm (<28 weeks)	11 (10)	5 (10)
Very preterm (28–32 weeks)	29 (27)	13 (27)
Moderate to late preterm (32–<37 weeks)	14 (13)	8 (17)
Term >37 weeks	55 (51)	22 (46)
Birthweight		
<10th percentile for gestational age [34]	23 (21)	12 (25)
Preconceptual risk factors [31]		
Maternal thyroid disease (hypo and hyperthyroidism)	10 (9)	5 (10)
Sibling with disability	16 (15)	3 (6)
Antenatal risk factors [31]		
In vitro fertilisation (IVF) conception	15 (14)	4 (8)
Congenital anomalies ^a^		
Cerebral ^b^	15 (14)	8 (17)
Cardiac	10 (9)	9 (19)
Musculoskeletal	17 (16)	5 (10)
Genitourinary	3 (3)	2 (4)
Digestive system	11 (10)	4 (8)
At least 1 congenital anomaly ^c^	42 (39)	20 (42)
Chorioamnionitis	7 (6)	2 (4)
Preeclampsia	15 (14)	7 (15)
Intrauterine growth restriction	21 (19)	11 (23)
Maternal infection during pregnancy (confirmed)	23 (21)	10 (21)
Multiple birth	12 (11)	6 (13)
Intrapartum risk factors [31]		
Confirmed hypoxic ischemic encephalopathy	11(10)	8 (17)
Meconium aspiration	2 (2)	0 (0)
Emergency caesarean section	53 (49)	30 (63)
Other instrumental delivery (non-emergency caesarean section, other instrumental delivery)	12 (11)	5 (10)
Neonatal and post neonatal risk factors [31]		
Presence of neonatal seizures	15 (14)	9 (19)
Respiratory Distress Syndrome	61 (56)	28 (58)
Neonate hypoglycaemia	26 (24)	12 (48)
Perinatal infection (up to 1 month of age)	9 (8)	3 (6)
Post neonatal infection (after 1 month of age)	7 (6)	3 (6)
Jaundice	46 (42)	23 (48)
Perinatal stroke (up to 1 month of age)	9 (8)	7 (15)
Post neonatal stroke (after 1 month of age)	3 (3)	3 (6)

Abbreviations: CP, cerebral palsy. ^a^ Congenital anomalies included major congenital anomalies requiring intervention confirmed in clinic report, e.g., cardiac surgery or inguinal hernia surgery; ^b^ Cerebral congenital anomalies include: microcephaly, hydrocephaly, cerebral cysts and corpus callosum anomalies. ^c^ Total number of infants who had at least one congenital anomaly.

**Table 3:** Age of diagnosis.

Diagnosis Process Stage	Newborn Detectable Risks (n = 76) ^a^			Infant Detectable Risks (n = 33)			All Infants (n = 109)		
	n	Age, Months		n	Age, Months		n	Age, Months	
		Mean (SD)	Median (Range)		Mean (SD)	Median (Range)		Mean (SD)	Median (Range)
Any clinical service suspected CP	53	3.6 (1.1)	4(0–7)	9	9.0 (2.7)	10 (5–12)	62	4.4(2.3)	4 (0–12)
CP or high risk of CP diagnosis at first clinic appointment	53	6.0 (2.6)	6 (0–13)	9	9.8 (2.7)	10 (5–13)	62	6.6 (3.0)	6 (0–13)
CP confirmed	40	8.1 (4.2)	7 (5–19)	8	10.6 (1.9)	10.5 (8–13)	48	8.5 (4.1)	8 (2–19)

Abbreviations: CP, cerebral palsy; n = number; SD, standard deviation. ^a^ n = 6 infants in the newborn detectable risks group did not have identified risks requiring follow up at birth but were assessed in time to have the General Movements Assessment video completed.

**Table 4:** Diagnostic Outcome.

	Newborn Detectable Risks, n (%) n = 76	Infant Detectable Risks, n (%) n = 33	All Infants, n (%) n = 109
CP	40 (53)	8 (24)	48 (44)
High risk of CP	13 (17)	1 (3)	14 (13)
No CP, other diagnosis ^a,b^	14 (18)	9 (27)	23 (21)
No CP, no concerns	8 (11)	14 (42)	22 (20)
Lost to follow up	1 (1)	1 (3)	2 (2)

Abbreviations: CP, cerebral palsy; n = number. ^a^ Other diagnoses suspected or confirmed: Autism Spectrum Disorder; congenital hypothyroidism; external hydrocephalus; global developmental delay (n = 3); language delay; meningoencephalitis; motor delay; persistent toe walking; plagiocephaly and torticollis; primary connective tissue disorders; genetic disorders not associated with CP. ^b^ Includes n = 1 infant with CP diagnosis revoked.

**Table 5:** Cerebral palsy-specific diagnostic tests completed ^a^.

	All Infants			CP		
	Newborn Detectable Risks, n (%) n = 76	Infant Detectable Risks, n (%) n = 33	Total, n (%) n = 109	Newborn Detectable Risks, n (%) n = 40	Infant Detectable Risks, n (%) n = 8	Total, n (%) n = 48
General Movements Assessment Writhing Period (^a^ recommendation 3.1)						
Administered externally prior to clinic	42 (55)	NA	42 (39)	21 (53)	NA	21 (44)
General Movements Assessment Fidgety Period (^a^ recommendation 3.1)						
Administered externally prior to clinic	70 (92)	NA	70 (64)	38 (95)	NA	38 (79)
Administered at clinic	6 (8)	NA	6 (6)	2 (5)	NA	2 (4)
No GMA, infant too old	0 (0)	NA	33 (30)	0 (0)	8 (100)	8 (17)
Neuroimaging (^a^ recommendation 4.2)						
Neuroimaging conducted prior to clinic						
CUS only	25 (33)	14 (42)	39 (36)	11 (28)	3 (38)	14 (29)
MRI only	29 (38)	5 (15)	34 (31)	20 (50)	2 (25)	22 (46)
CUS + MRI	19 (25)	0 (0)	19 (17)	9 (23)	0 (0)	9 (19)
No neuroimaging prior to clinic	3 (4)	14 (42)	17 (16)	0	3 (38)	3 (6)
HINE (^a^ recommendations 4.2, 6.1 and 7.1)						
Administered externally prior to clinic	46 (61)	11 (33)	57 (52)	21 (53)	2 (25)	23 (48)
First administration at clinic	30 (40)	22 (67)	51 (47)	18 (45)	6 (75)	24 (50)
HINE not administered	1 (1)	0 (0)	1 (1)	1 (3)	0 (0)	1 (2)

Abbreviations: CP, cerebral palsy; CUS, cranial ultrasound; GMA, General Movements Assessment; HINE, Hammersmith Infant Neurological Examination; MRI, magnetic resonance imaging; NA, not applicable. ^a^ Assessments based on recommendations 2.0 and 3.0–7.1 of the international clinical guideline for early and accurate detection of cerebral palsy [1].

## Supplementary Materials

The following are available online at https://www.mdpi.com/article/10.3390/brainsci11081074/s1, Table S1: Standards for Reporting Implementation Studies: the StaRI checklist, Table S2: Barriers, Facilitators with Corresponding Knowledge Translation Strategies of Early Diagnosis Clinic.
