# Supplementary material for: Age of Diagnosis, Fidelity and Acceptability of an Early Diagnosis Clinic for Cerebral Palsy: A Single Site Implementation Study
Source: Brain Sci. 2021 Aug 16;11(8):1074. doi: 10.3390/brainsci11081074 (PMC8391606; doi:10.3390/brainsci11081074)
Supplement: Supplementary file 1 [file brainsci-11-01074-s001.zip › brainsci-1303025 Table Supple.pdf]

Table S1. Standards for Reporting Implementation Studies: the StaRI checklist [1].

Checklist item		Reported on page #	Implementation Strategy	Reported on page #	Intervention
			"Implementation strategy" refers to how the intervention was implemented		"Intervention" refers to the healthcare or public health intervention that is being implemented.<br><br>International Clinical Guideline on the Early and Accurate Diagnosis of Cerebral Palsy
**Title and abstract**					
Title	**1**	Page 1	Identification as an implementation study, and description of the methodology in the title and/or keywords		
Abstract	**2**	Page 1	Identification as an implementation study, including a description of the implementation strategy to be tested, the evidence-based intervention being implemented, and defining the key implementation and health outcomes.		
**Introduction**					
Introduction	**3**	Page 1	Description of the problem, challenge or deficiency in healthcare or public health that the intervention being implemented aims to address.		
Rationale	**4**	Page 2 + Supp Table S2	The scientific background and rationale for the implementation strategy (including any underpinning theory/framework/model, how it is expected to achieve its effects and any pilot work).	Page 1-2 – Internatio nal Clinical Guideline	The scientific background and rationale for the intervention being implemented (including evidence about its effectiveness and how it is expected to achieve its effects).
Aims and objectives	**5**	Page 2	The aims of the study, differentiating between implementation objectives and any intervention objectives.		
**Methods: description**					

Design	**6**	Page 3	The design and key features of the evaluation, (cross referencing to any appropriate methodology reporting standards) and any changes to study protocol, with reasons		
Context	**7**	Page 5 (site)	The context in which the intervention was implemented. (Consider social, economic, policy, healthcare, organisational barriers and facilitators that might influence implementation elsewhere).		
Targeted 'sites'	**8**	Page 4 (site)	The characteristics of the targeted 'site(s)' (e.g locations/personnel/resources etc.) for implementation and any eligibility criteria.	Page 4	The population targeted by the intervention and any eligibility criteria.
Description	**9**	Page 3 + Supp Table S2	A description of the implementation strategy	Table 1	A description of the intervention
Sub-groups	**10**	N/A	Any sub-groups recruited for additional research tasks, and/or nested studies are described		
**Methods: evaluation**					
Outcomes	**11**	Page 5	Defined pre-specified primary and other outcome(s) of the implementation strategy, and how they were assessed. Document any pre-determined targets	Page 5	Defined pre-specified primary and other outcome(s) of the intervention (if assessed), and how they were assessed. Document any pre-determined targets
Process evaluation	**12**	Supp Table S2	Process evaluation objectives and outcomes related to the mechanism by which the strategy is expected to work		
Economic evaluation	**13**	N/A	Methods for resource use, costs, economic outcomes and analysis for the implementation strategy	N/A	Methods for resource use, costs, economic outcomes and analysis for the intervention
Sample size	**14**	Page 3	Rationale for sample sizes (including sample size calculations, budgetary constraints, practical considerations, data saturation, as appropriate)		
Analysis	**15**	Page 5	Methods of analysis (with reasons for that choice)		
Sub-group analyses	**16**	N/A	Any a priori sub-group analyses (e.g. between different sites in a multicentre study, different clinical or demographic populations), and sub-groups recruited to specific nested research tasks		

**Results**					
Characteristics	**17**	Page 4 (site)	Proportion recruited and characteristics of the recipient population for the implementation strategy	Page 5 + Table 2	Proportion recruited and characteristics (if appropriate) of the recipient population for the intervention
Outcomes	**18**	Page 6 + Table 3,4 &5	Primary and other outcome(s) of the implementation strategy	Page 6 + Table 3,4 &5	Primary and other outcome(s) of the Intervention (if assessed)
Process outcomes	**19**	Supp Table S2	Process data related to the implementation strategy mapped to the mechanism by which the strategy is expected to work		
Economic evaluation	**20**	N/A	Resource use, costs, economic outcomes and analysis for the implementation strategy	N/A	Resource use, costs, economic outcomes and analysis for the intervention
Sub-group analyses	**21**	N/A	Representativeness and outcomes of subgroups including those recruited to specific research tasks		
Fidelity/ adaptation	**22**	Page 7 + Supp Table S2	Fidelity to implementation strategy as planned and adaptation to suit context and preferences	Page 7	Fidelity to delivering the core components of intervention (where measured)
Contextual changes	**23**	N/A	Contextual changes (if any) which may have affected outcomes		
Harms	**24**	Page 9	All important harms or unintended effects in each group		
**Discussion**					
Structured discussion	**25**	Page 9	Summary of findings, strengths and limitations, comparisons with other studies, conclusions and implications		
Implications	**26**	Pages 9-11	Discussion of policy, practice and/or research implications of the implementation strategy (specifically including scalability)	Pages 9-11	Discussion of policy, practice and/or research implications of the intervention (specifically including sustainability)

General			
Statements	**27**	Page 12	Include statement(s) on regulatory approvals (including, as appropriate, ethical approval, confidential use of routine data, governance approval), trial/study registration (availability of protocol), funding and conflicts of interest

Table S2. Barriers, Facilitators with Corresponding Knowledge Translation Strategies of Early Diagnosis Clinic.

Barriers and facilitators to implementation of guideline	Knowledge translation strategies used in design of Early Diagnosis Clinic	Adaptations of knowledge translation interventions for study	How knowledge translation strategy is expected to achieve its effects
**Barrier - Practitioner attitudes**<br>Healthcare professionals' attitude at major health institutions in NSW that CP could not accurately be diagnosed under 12 months of age	Targeted **practitioner attitudes** by explicit training of predictive value of GMA and HINE (tools to predict CP)<br>Feedback loop to referring clinicians of diagnostic outcome and intervention pathways for infants referred to Early Diagnosis Clinic<br>Neurologists from NSW Health targeted for referrals	Ensured referral pathways and early intervention services available for infants identified with CP or high risk of CP for referring practitioners to trust something could be done for referred infants	**Practitioner perception, attitude and beliefs** about evidence-based practice are predictors of evidence-based practice use in health practitioners [2]
Healthcare professionals concerned about upsetting parents unnecessarily when they feel nothing can be done for infants	Improved **guideline awareness** used explicit teaching of guidelines at the time of publication e.g. presented at major teaching hospital Grand Rounds and explicit education to targeted audiences at industry conferences		Awareness of the existence of a clinical guideline, including guideline content, affects implementation [3]
**Barriers**<br>1. Referrers unsure if a diagnosis of CP should be made for borderline cases<br>2. Referrers unsure of which babies to fast track for CP diagnosis<br>3. Referrers unsure or untrained in use of best practice tools	1. **Knowledge brokers** were identified and trained as staff on the clinic team to feedback and coach referrers regarding outcome of diagnosis.<br>2. **Knowledge brokers** used early diagnosis evidence to inform policy and decision makers within both collaborating institutions (Cerebral Palsy Alliance and NSW Health);	Existing **Community of Practice,** NSW GMA rater network reliability days were adapted to include discussion about early detection of CP rather than just GMA video scoring	**Knowledge brokers** work one on one with decision makers to facilitate evidence-informed decision making. [4] It is likely that knowledge brokers need to work in conjunction with a **community of practice** to be successful [4]. In order for knowledge brokerage to be most effective knowledge needs to be given to the right decision maker.

	3. Clinic staff linked with members of existing community of practice, the NSW GMA rater network, who were likely to be referrers to clinic and key decision makers		
**Barrier: Identified Gaps in Healthcare Service**<br>1. Gap: No service to capture infants with risk for CP who were not seen in NICU follow up clinics<br>2. Gap: No service which focused on specifically on early diagnosis of CP<br>3. Gap: personnel to staff clinic	1. Creation of a new service to fill gap<br>2. **Opinion Leaders:** A team of multi-disciplinary opinion leaders targeted to staff the clinic and develop the Early Diagnosis Clinic to meet the identified service gap<br>3. **Mentoring** of clinic staff by industry experts and mentoring clinic staff of clinicians in the wider CP field.	Utilisation of opinion leaders to make use of GMA standard of care in NSW NICU follow up clinics [5,6] had previously been successful, but a new clinic was required to fill service gap.	Use of **opinion leader strategy** as a part of a multifaceted implementation strategy increases the evidence to practice uptake by 10% (median risk difference = +0.10) [7]
**Healthcare Barrier**<br>1. Healthcare clinicians did not believe parents who had concern about their child's development or felt a "wait and see approach" was required to see if an infant should be referred for diagnosis process	1. A top down (medical referral) and bottom up [8] (parent referral to clinic) would be more likely to capture infants, particularly in the infant detectable risks group. Most parents suspect CP before a diagnosis is made [9]	Parent referral accepted (with supporting medical referral for Medicare billing)	Patient (parent)-mediated knowledge translation strategies improve population health outcomes [10]
**System Facilitator**<br>1. Research active implementing organisations (Cerebral Palsy Alliance and NSW Health)	1. Best use made of current **research active** culture at Cerebral Palsy Alliance and NSW Health (implementing organisations)<br>2. Continued investment in research active culture at implementing organisations	Employed research clinicians on clinic team	Involvement in research activities are a significant predictor of self-reported research evidence use in health practitioners [2].<br><br>Investment in research culture in an organisation is associated with improved outcomes (mortality), organisational efficiency and patient satisfaction [11].
**Healthcare Barrier**<br>6 months wait time to see neurologist in public health care system in Southwest Sydney	1. **Employment of neurologist** from NSW Health to lead Early Diagnosis Clinic team. Research work included in position		A responsive service with short wait time will encourage clinicians to refer to this service

Abbreviations: CP, cerebral palsy; GMA; General Movements Assessment; HINE, Hammersmith Infant Neurological Examination; NICU, Neonatal Intensive Care Unit; NSW, New South Wales. [a] Table based on Standards for Reporting Implementation Studies (StaRI) [1].

References

1. Pinnock, H.; Barwick, M.; Carpenter, C.R.; Eldridge, S.; Grandes, G.; Griffiths, C.J.; Rycroft-Malone, J.; Meissner, P.; Murray, E.; Patel, A.; et al. Standards for Reporting Implementation Studies (StaRI) Statement. *BMJ* **2017**, *356*, doi:10.1136/bmj.i6795.
2. Lizarondo, L.; Grimmer-Somers, K.; Kumar, S. A systematic review of the individual determinants of research evidence use in allied health. *J. Multidiscip. Healthc.* **2011**, *4*, 261–272, doi:10.2147/JMDH.S23144.
3. Francke, A.L.; Smit, M.C.; de Veer, A.J.E.; Mistiaen, P. Factors influencing the implementation of clinical guidelines for health care professionals: A systematic meta-review. *BMC Med. Inform. Decis. Mak.* **2008**, *8*, 38–38, doi:10.1186/1472-6947-8-38.
4. Dobbins, M.; Hanna, S.; Ciliska, D.; Manske, S.; Cameron, R.; Mercer, S.; O'Mara, L.; Decorby, K.; Robeson, P. A randomized controlled trial evaluating the impact of knowledge translation and exchange strategies. *Implementat. Sci.* **2009**, *4*, doi:10.1186/1748-5908-4-61.
5. Goyen, T.-A.; Morgan, C.; Crowle, C.; Hardman, C.; Day, R.; Novak, I.; Badawi, N. Sensitivity and specificity of general movements assessment for detecting cerebral palsy in an Australian context: 2-year outcomes. *J. Paediatr. Child. Health* **2020**, *56*, 1414–1418, doi:10.1111/jpc.14953.
6. Crowle, C.; Badawi, N.; Walker, K.; Novak, I. General Movements Assessment of infants in the neonatal intensive care unit following surgery. *J. Paediatr. Child. Health* **2015**, *51*, 1007–1011, doi:10.1111/jpc.12886.
7. Flodgren, G.; Parmelli, E.; Doumit, G.; Gattellari, M.; O'Brien, M.A.; Grimshaw, J.; Eccles, M.P. Local opinion leaders: Effects on professional practice and health care outcomes. *Cochrane Database Syst. Rev.* **2011**, *8*, CD000125, doi:10.1002/14651858.CD000125.pub4.
8. Proctor, E.; Landsverk, J.; Aarons, G.; Chambers, D.; Glisson, C.; Mittman, B. Implementation Research in Mental Health Services: An Emerging Science with Conceptual, Methodological, and Training challenges. *Adm. Policy Ment. Health Ment. Health Serv. Res.* **2009**, *36*, 24–34, doi:10.1007/s10488-008-0197-4.
9. Baird, G.; McConachie, H.; Scrutton, D. Parents' perceptions of disclosure of the diagnosis of cerebral palsy. *Arch. Dis. Child.* **2000**, *83*, 475–480, doi:10.1136/adc.83.6.475.
10. Gagliardi, A.R.; Légaré, F.; Brouwers, M.C.; Webster, F.; Badley, E.; Straus, S. Patient-mediated knowledge translation (PKT) interventions for clinical encounters: A systematic review. *Implement. Sci.* **2016**, *11*, 26–26, doi:10.1186/s13012-016-0389-3.
11. Harding, K.; Lynch, L.; Porter, J.; Taylor, N.F. Organisational benefits of a strong research culture in a health service: A systematic review. *Aust. Health Rev.* **2016**, *41*, 45–53, doi:10.1071/AH15180.
